# Biallelic variants in *ZNF142* lead to a syndromic neurodevelopmental disorder

**DOI:** 10.1111/cge.14165

**Published:** 2022-06-08

**Authors:** Maria B. Christensen, Amanda M. Levy, Nazanin A. Mohammadi, Marcello Niceta, Rauan Kaiyrzhanov, Maria Lisa Dentici, Chadi Al Alam, Viola Alesi, Valérie Benoit, Kailash P. Bhatia, Tatjana Bierhals, Christian M. Boßelmann, Julien Buratti, Bert Callewaert, Berten Ceulemans, Perrine Charles, Matthias De Wachter, Mohammadreza Dehghani, Erika D'haenens, Martine Doco‐Fenzy, Michaela Geßner, Cyrielle Gobert, Ulviyya Guliyeva, Tobias B. Haack, Trine B. Hammer, Tilman Heinrich, Maja Hempel, Theresia Herget, Ute Hoffmann, Judit Horvath, Henry Houlden, Boris Keren, Christina Kresge, Candy Kumps, Damien Lederer, Alban Lermine, Francesca Magrinelli, Reza Maroofian, Mohammad Yahya Vahidi Mehrjardi, Mahdiyeh Moudi, Amelie J. Müller, Anna J. Oostra, Beth A. Pletcher, David Ros‐Pardo, Shanika Samarasekera, Marco Tartaglia, Kristof Van Schil, Julie Vogt, Evangeline Wassmer, Juliane Winkelmann, Maha S. Zaki, Michael Zech, Holger Lerche, Francesca Clementina Radio, Paulino Gomez‐Puertas, Rikke S. Møller, Zeynep Tümer

**Affiliations:** ^1^ Department of Clinical Genetics Copenhagen University Hospital Copenhagen Denmark; ^2^ Kennedy Center, Department of Clinical Genetics Copenhagen University Hospital Copenhagen Denmark; ^3^ Department of Epilepsy Genetics and Personalized Treatment The Danish Epilepsy Centre Dianalund Denmark; ^4^ Department of Regional Health Research University of Southern Denmark Odense Denmark; ^5^ Genetics and Rare Diseases Research Division Ospedale Pediatrico Bambino Gesù, IRCCS Rome Italy; ^6^ Department of Neuromuscular Disorders University College London Institute of Neurology London UK; ^7^ Medical Genetics Unit, Academic Department of Pediatrics Bambino Gesù Children's Hospital, IRCCS Rome Italy; ^8^ Pediatric Neurology department American center for Psychiatry and Neurology Al Ain United Arab Emirates; ^9^ Pediatric Neurology department Haykel Hospital El Koura Lebanon; ^10^ Translational Cytogenomics Research Unit Ospedale Pediatrico Bambino Gesù, IRCCS Rome Italy; ^11^ IPG Centre for Human Genetics Charleroi Belgium; ^12^ Department of Clinical and Movement Neurosciences, UCL Queen Square Institute of Neurology University College London London UK; ^13^ Institute of Human Genetics University Medical Center Hamburg‐Eppendorf Hamburg Germany; ^14^ Department of Neurology and Epileptology, Hertie‐Institute for Clinical Brain Research University of Tübingen Tübingen Germany; ^15^ Department of Medical Genetics, Pitié‐Salpêtrière Hospital AP‐HP, Sorbonne Université Paris France; ^16^ Center for Medical Genetics Ghent University Hospital Ghent Belgium; ^17^ Department of Biomolecular Medicine Ghent University Ghent Belgium; ^18^ Department of Pediatric Neurology, Antwerp University Hospital University of Antwerp Edegem Belgium; ^19^ Medical Genetics Research Center Shahid Sadoughi University of Medical Sciences Yazd Iran; ^20^ SFR CAP SANTE HMB2 CHU Reims Reims France; ^21^ CHU de Nantes, service de génétique médicale Nantes France; ^22^ KfH‐Board of Trustees for Dialysis and Kidney Transplantation (KfH‐Kuratorium für Dialyse und Nierentransplantation e.V.) Neu Isenburg Germany; ^23^ Neuropediatric department Centre Hospitalier Neurologique William Lennox Ottignies Belgium; ^24^ Department of Pediatrics MediClub Hospital Baku Azerbaijan; ^25^ Institute of Medical Genetics and Applied Genomics University of Tübingen Tübingen Germany; ^26^ Centre for Rare Diseases University of Tübingen Tübingen Germany; ^27^ MVZ Humangenetik und Molekularpathologie GmbH Rostock Germany; ^28^ St. Franziskus‐Hospital Münster Germany; ^29^ Institute of Human Genetics University of Münster Münster Germany; ^30^ Department of Pediatrics Rutgers New Jersey Medical School Newark New Jersey USA; ^31^ LBBMS SeqOIA AP‐HP Paris France; ^32^ Department of Genetics Shahid Sadoughi University of Medical Sciences Yazd Iran; ^33^ Neuropediatric department Ghent University Hospital Ghent Belgium; ^34^ Centre for Developmental disorders University Hospital Ghent Ghent Belgium; ^35^ Molecular Modeling Group Centro de Biología Molecular Severo Ochoa, CBMSO (CSIC‐UAM) Madrid Spain; ^36^ Neurology Department Queen Elizabeth Hospital Birmingham UK; ^37^ Department of Medical Genetics, Antwerp University Hospital University of Antwerp Edegem Belgium; ^38^ West Midlands Regional Genetics Service Birmingham Women's and Children's Hospital Birmingham UK; ^39^ Neurology Department Birmingham Women and Children's Hospital Birmingham UK; ^40^ Institute of Health and Neurodevelopment Aston University Birmingham UK; ^41^ Institute of Human Genetics, School of Medicine Technical University of Munich Munich Germany; ^42^ Institute of Neurogenomics Helmholtz Zentrum München Munich Germany; ^43^ Clinical Genetics Department, Human Genetics and Genome Research Institute National Research Centre Cairo Egypt; ^44^ Genetics Department Armed Forces College of Medicine (AFCM) Cairo Egypt; ^45^ Department of Clinical Medicine, Faculty of Health and Medical Sciences University of Copenhagen Copenhagen Denmark

**Keywords:** epilepsy, intellectual disability, language impairement, movement disorder, neurodevelopmental disorder, ZNF142

## Abstract

Biallelic variants of the gene encoding for the zinc‐finger protein 142 (*ZNF142*) have recently been associated with intellectual disability (ID), speech impairment, seizures, and movement disorders in nine individuals from five families. In this study, we obtained phenotype and genotype information of 26 further individuals from 16 families. Among the 27 different *ZNF142* variants identified in the total of 35 individuals only four were missense. Missense variants may give a milder phenotype by changing the local structure of ZF motifs as suggested by protein modeling; but this correlation should be validated in larger cohorts and pathogenicity of the missense variants should be investigated with functional studies. Clinical features of the 35 individuals suggest that biallelic *ZNF142* variants lead to a syndromic neurodevelopmental disorder with mild to moderate ID, varying degrees of delay in language and gross motor development, early onset seizures, hypotonia, behavioral features, movement disorders, and facial dysmorphism. The differences in symptom frequencies observed in the unpublished individuals compared to those of published, and recognition of previously underemphasized facial features are likely to be due to the small sizes of the previous cohorts, which underlines the importance of larger cohorts for the phenotype descriptions of rare genetic disorders.

## Graphical abstract

Zinc‐finger protein 142, encoded by *ZNF142*, belongs to a family transcriptional repressors that recruit chromatin remodeling proteins to DNA. They are widely expressed and are important for numerous processes such as development, cell differentiation and proliferation, and apoptosis. Zn^2+^ ions (gray) and three modellable missense variants (yellow) are depicted as spheres.

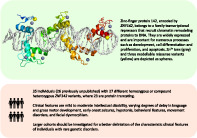

## 1 INTRODUCTION

The zinc‐finger protein (ZNF) superfamily is one of the largest groups of mammalian transcription factors and various diseases have been linked to pathogenic variants in ZNF genes. The widely expressed KRAB‐ZNF (Krüppel‐associated box) proteins belong to the classical ZNF subgroup (C_2_H_2_‐ZNFs), in which proper conformation of the zinc‐finger (ZF) motif is stabilized by two cysteine and two histidine residues coordinating a Zn^2+^ ion.^1^ The KRAB domains function as transcriptional repressors and exert their effect through recruiting chromatin remodeling proteins to DNA‐binding sites.^2^, ^3^ The KRAB‐ZNF proteins are involved in embryonic development, cell differentiation and proliferation, apoptosis, neoplastic transformation, and cell cycle regulation.^4^, ^5^ Individual KRAB‐ZNFs have been associated with regulation of brain and head size in mice and humans, respectively,^6^ as well as neural differentiation and proliferation.^7^, ^8^

Several genes encoding KRAB‐ZNF proteins have been associated with neurodevelopmental disorders (NDDs). Haploinsufficiency of four KRAB‐ZNF genes (*ZNF302*, *ZNF181*, *ZNF599*, and *ZNF30*) was proposed as the cause of the 19q13.11‐microdeletion syndrome, which is characterized by developmental delay, speech disturbances, growth retardation, microcephaly, dysmorphic facies, signs of ectodermal dysplasia, and hypospadias.^9^ Biallelic variants in *ZNF526* were identified in five individuals with epilepsy, microcephaly, and bilateral cataract,^10^ and two siblings with nonsyndromic NDD had biallelic *ZNF589* variants.^11^ An Xp11.2p11.3 microduplication encompassing *ZNF81* and *ZNF182* was found in an individual with NDD, autistic features, and delayed growth and speech.^12^ Variants in several other KRAB‐ZNF genes (*ZNF41, ZNF81, ZNF148, ZNF673*, and *ZNF674*) were associated with X‐linked NDD,^13^, ^14^, ^15^, ^16^ while others were implicated in autism spectrum disorder (ASD), schizophrenia, major depression, bipolar affective disorder, and Parkinson's disease.^1^, ^17^, ^18^, ^19^

Pathogenic biallelic variants of the KRAB‐ZNF gene *ZNF142* was first reported in 2019 in seven individuals from four families,^20^ and subsequently in two siblings.^21^ In the current work, we describe the clinical phenotype of 26 previously unpublished individuals with biallelic *ZNF142* variants from 16 unrelated families and fine‐tune the clinical features and their frequencies of this rare NDD.

## 2 METHODS

**FIGURE 1:**
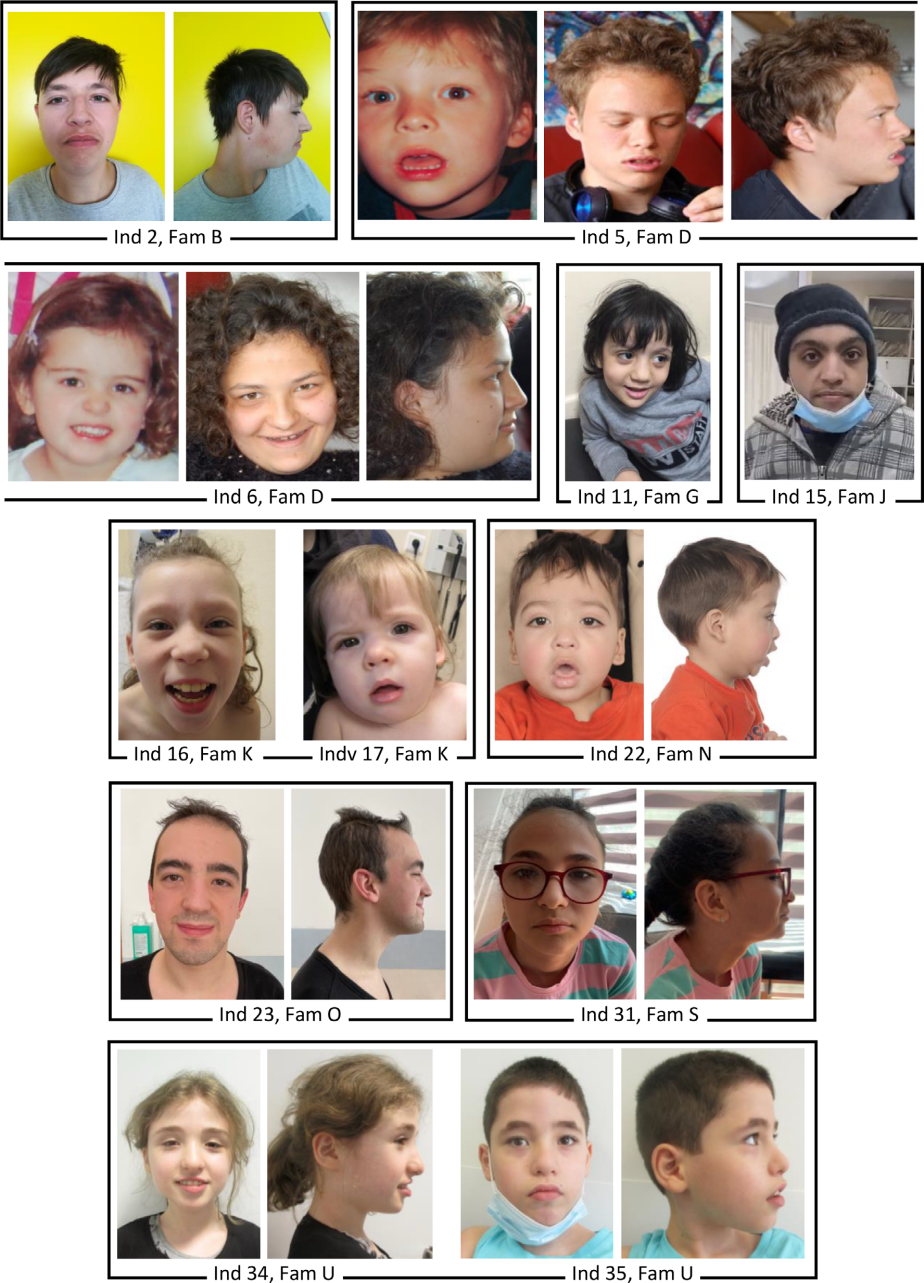
Facial photographs of the individuals carrying biallelic pathogenic variants in *ZNF142*. Age of the individuals (ind) when photographed: Ind 2, 17 years; Ind 5, 17 years in middle and right photographs, age unknown in left photograph; Ind 6, 14 years in middle and right photographs, age unknown in left photograph; Ind 11, 5 years; Ind 12, 20 years; Ind 16, 10 years; Ind 17, 18 months; Ind 22, 3 years; Ind 23, 22 years; Ind 31, 12 years; Ind 34, 15 years; Ind 35, 9 years. Fam, Family [Colour figure can be viewed at wileyonlinelibrary.com]

Twenty‐six individuals from 16 families were included through GeneMatcher (www.genematcher.org)^22^ or ERN‐ITHACA networks (www.ern-ithaca.eu). Nine individuals from five families were previously published: Individuals 3, 4, 21, 27, 28, 29, and 30^20^ and individuals 9 and 10.^21^ The phenotypes of the individuals described by Khan et al.^20^ were revised and no disease progression was observed. The clinical features and the cognitive function of the new patients were assessed by the local clinicians. The facial features of the individuals for whom a clinical picture was available (Figure 1) were evaluated independently by three dysmorphologists from our group (Table S1). When a formal cognitive test was performed, the DSM‐IV scale was used for the subclassification of the severity of intellectual disability (ID). It was not possible to attain a complete phenotypic profile on all individuals included in the current study. All families gave consent to publication of the clinical and genetic information, and inclusion of the clinical pictures.

Variants in *ZNF142* were detected using next generation sequencing based technologies (clinical exome or whole genome sequencing) (Table S2). All variants were described using the *ZNF142* transcript NM_001105537.2 (GRCh37/hg19) according to Human Genome Variation Society recommendations (https://varnomen.hgvs.org/) and confirmed by VariantValidator (https://variantvalidator.org/). To review the presence of the variants in the control populations, the Genome Aggregation Database (gnomAD v.2.1.1; https://gnomad.broadinstitute.org/) was used. For the missense variants, splicing effects were predicted using the splice variant prediction tool, SpliceAI (https://github.com/Illumina/SpliceAI). To assess whether the nonsense variants would escape nonsense mediated decay (NMD), the 55‐bp rule was employed, that is, premature termination codons (PTCs) must be a maximum of 55 bp upstream of the last exon‐exon junction to escape NMD.^23^ Furthermore, the NMDEscPredictor tool (https://nmdprediction.shinyapps.io/nmdescpredictor/) was used to assess the frameshift variants' ability to escape NMD. combined annotation dependent depletion (CADD), https://cadd.gs.washington.edu/) scores were used to evaluate deleteriousness of all the variants, and for missense variants REVEL (Rare Exome Variant Ensemble Learner, https://sites.google.com/site/revelgenomics) scores were also calculated.

Structural modeling of ZF motifs 18 to 26 located in the ZNF142 C‐terminal (UniprotKB id: P52746) was performed using the crystal structure of ZF motifs 5–8 of human CCCTC‐binding factor in complex with DNA (PDB id: 5K5I^24^) and of ZF motifs 2–11 of mouse ZFP568 in complex with DNA (PDB id: 5WJQ^25^) as templates. Multiple sequence alignment of ZF motifs was obtained using MUSCLE in EMBL‐EBI facilities.^26^ Figures were generated using the PyMOL Molecular Graphics System (https://pymol.org/2/).

## 3 RESULTS

### 3.1 Clinical findings

The frequencies of the clinical features are presented in Table 1 and the details in Table S2. The female‐to‐male ratio was 3:2 and the age at the time of the last clinical examination was 3–42 years.

**TABLE 1:** Frequency of the most common clinical features

	Present study*n* = 26		Published^a^ *n* = 9		Total *n* = 35	
Feature	Ratio	%	Ratio	%	Ratio	%
Intellectual disability	23/26	89%	8/9	89%	31/35	89%
Language impairment	26/26	100%	9/9	100%	35/35	100%
Delay in motor milestones	22/26	85%	6/9	67%	28/35	80%
Seizures	17/26	65%	7/9	78%	24/35	69%
Behavioral challenges	12/22	55%	4/9	44%	16/31	52%
Hypotonia^b^	10/19	52%	3/9	33%	13/28	46%
Movement disorders	9/25	36%	5/9	56%	14/34	43%
Dystonia	5/25	20%	3/9	33%	8/34	24%
Tremor	4/25	16%	4/9	44%	8/34	24%
Ataxia	7/25	28%	4/9	44%	11/34	32%

*Note*: *n*, number of individuals in each cohort.

^a^ Previously reported by Khan et al. 2019 (20) and Kameyama et al. 2021 (21).

^b^ Facial hypotonia not included.

Mild and moderate ID was described in 10 and 15 individuals, respectively. One individual had moderate–severe ID (individual 26), four individuals had severe ID (individuals 7, 9, 19, and 30), and one showed global developmental delay (GDD) (individual 22). Formal cognitive testing was performed in 18 individuals (Table 1). Three individuals were described as having low average IQ (individuals 12, 13, and 27, and the IQ scores are in Table S2), and one 3‐year‐old child was clinically evaluated as intellectually normal, but no cognitive testing was performed (individual 14).

The severity of language impairment varied from no acquired speech to extensive but not age‐appropriate vocabulary. The expressive language was most affected, while the receptive language generally was described as more age‐appropriate. The language skills of most individuals were limited with an active vocabulary of only a few words. Individuals 18, 19, and 32 were described as speaking slowly.

Twenty‐eight individuals (28/35) were delayed in gross motor function, defined by gaining individual ambulatory function later than 18 months of age. Walking could be achieved later in childhood as exemplified in individuals 34 and 35, who started walking at age 3 and 6 years, respectively.

Eleven individuals did not present with any behavioral challenges, while one or more psychiatric/behavioral features were reported in 16 individuals (16/27, 60%). These included autistic behavior or limited socialization (*n* = 8), attention deficit hyperactivity disorder (ADHD), or attention deficiency disorder (ADD) (*n* = 8); and aggressivity and anxiety were reported in a few individuals. For all the individuals, the autistic behavior and ADD/ADHD were assessed clinically without formal testing and in several cases the features were subclinical. Information about behavioral challenges was not available or not mentioned in the previous publications for the remaining eight individuals.

One or more movement disorders, such as dystonia, ataxia, and tremor, were reported in 14 individuals. Severity of the different movement disorders varied greatly. Dystonia (*n* = 8) was described as ranging from possible or mild to generalized and persistent. Tremors (*n* = 8) were described as either action‐induced or constant and involving only the hands, head, chin, and/or upper limbs, or described as generalized. Ataxia (*n* = 11) was both described as constant, such as mild ataxic or broad‐based gait, or periodic, such as head nodding on concentration or ‘attacks’ with ataxia and ambulatory difficulties. One individual had tremor with onset at age 41, potentially due to drug side‐effect (individual 24), and 19 individuals did not present with any movement disorders.

Hypotonia was reported in 13 individuals (46%). Peripheral hypertonia was described only in two individuals (individuals 17 and 27), and one individual who currently has normal tonus had hypertonia in infancy (individual 14). Seven individuals had normal muscle tonus.

Twenty‐four individuals (24/35, 69%) had experienced at least one seizure, and the age of onset was in infancy or early childhood (before age 2 years) in most of the individuals (*n* = 20), and the majority had more than one seizure (21/23). The first seizure was afebrile in six individuals and fever‐induced in 10 individuals, and in the latter group, five individuals subsequently developed afebrile seizures, triggered by factors such as fatigue, sleep deprivation, and cessation of antiepileptic drugs (AED). Information about the first seizure was not available for eight individuals. Epilepsy was observed in 19 individuals and the most prevalent seizure type was generalized tonic–clonic seizures (*n* = 13) followed by focal (*n* = 6) and absence (*n* = 5) seizures. Myoclonic and unspecific seizures were observed in single individuals, and six individuals had status epilepticus. Electroencephalogram (EEG) recordings were available for 19 individuals, and except for one individual (individual 7), recordings were performed interictally. Twelve individuals had normal EEGs. For the seven individuals with abnormal EEGs generalized background slowing was the only recurrent abnormal pattern observed in four individuals. Epileptic activity was not observed in any of the interictal EEGs.

In 22 individuals with brain imaging, no gross structural changes were observed. In six individuals, nonspecific changes such as thinning of corpus callosum and delayed myelination were recurrent. As all the individuals were older than 3 years of age, observation of delayed myelination was not attributed to the young age. Cerebral imaging was not carried out in seven individuals.

The most common facial features of the current cohort as reported by the referring clinicians included thick lips and eyebrows, epicanthus, and broad nasal bridge, but did not indicate a recognizable facial gestalt. However, independent inspections of the clinical pictures of 12 individuals from the current cohort (Figure 1) and two individuals reported by Kamemaya et al.^21^ by three dysmorphologists revealed hypertelorism and wide nasal bridge, telecanthus, thick eyebrows with lateral sparseness, everted lower lip vermilion, and facial hypotonia in more than half of the 14 individuals (Table S1). Among the 35 individuals there were no recurrent skeletal manifestations or involvement of other large organs.

### 3.2 Genotype findings

**FIGURE 2:**
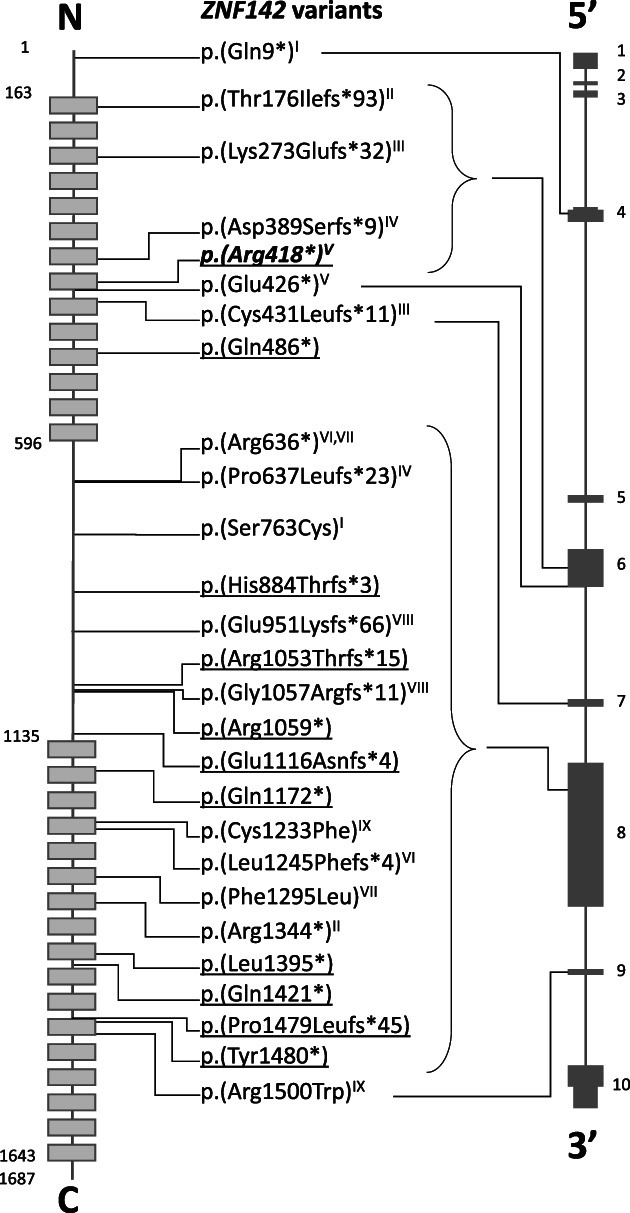
Schematic illustration of the location of each *ZNF142* variant at protein (left) and DNA (right) levels. (Left) Each light gray box represents one of the 31 ZF motifs. (Middle) Underlined text: Homozygous variant. Nonunderlined text: Compound heterozygous variant *in trans* with the variant with the same roman numeral. Underlined, bold, italic text: Detected as homozygous and compound heterozygous variant *in trans* with the variant with the same roman numeral; (Right) The dark gray boxes represent *ZNF142* exons. The narrow boxes represent the untranslated regions. N, N‐terminal; C, C‐terminal

Twenty‐seven different *ZNF142* variants (eight previously published) were identified in 35 individuals (nine previously published) from 21 families (Table 2 and Table S2, Figure 2). Twenty‐one individuals from 12 families were homozygous and 14 individuals from nine families were compound heterozygous for the *ZNF142* variants. For individual 23, who was homozygous for a single‐nucleotide deletion, the mother was heterozygous, while the father did not have the variant. The homozygosity was subsequently determined to be due to an 8.5 kb maternal uniparental disomy (UPD) including *ZNF142* using a single‐nucleotide polymorphism (SNP) array analysis, which also ruled out other clinically relevant structural rearrangements. A real‐time quantitative PCR (qPCR) assay also excluded a deletion involving *ZNF142*.

**TABLE 2:** Homozygous and compound heterozygous variants in *ZNF142* in 35 individuals

			Allele 1				Allele 2				
Fam	Ind^a^	Zygosity	Variant type	Variant description	Predicted protein	CADD score (#)	Variant type	Variant description	Predicted protein	CADD score (#)	References
A	1	Comp het	Non	c.25C > T	p.(Gln9*)	33	Mis	c.2288C > G	p.(Ser763Cys)	15.26 (0.055)	Present
B	2	Comp het	Fs	c.527del	p.(Thr176Ilefs*93)	32	Non	c.4030C > T	p.(Arg1344*)	39	Present
C	3,4	Comp het	Fs	c.817_818del	p.(Lys273Glufs*32)	32	Fs	c.1292del	p.(Cys431Leufs*11)	33	Khan^b^
D	5,6	Comp het	Fs	c.1165_1166del	p.(Asp389Serfs*9)	33	Fs	c.1910del	p.(Pro637Leufs*23)	22.7	Present
E	7,8	Hom	Non	c.1252C > T	p.(Arg418*)	38	Non	c.1252C > T	p.(Arg418*)	38	Present
F	9,10	Comp het	Non	c.1252C > T	p.(Arg418*)	38	Non	c.1274‐2A > G	p.(Glu426*)	34	Kameyama^c^
G	11	Hom	Non	c.1456C > T	p.(Gln486*)	37	Non	c.1456C > T	p.(Gln486*)	37	Present
H	12,13	Comp het	Non	c.1906C > T	p.(Arg636*)	34	Non	c.3735del	p.(Leu1245Phefs*4)	33	Present
I	14	Comp het	Non	c.1906C > T	p.(Arg636*)	34	Mis	c.3885C > A	p.(Phe1295Leu)	25.8 (0.266)	Present
J	15	Hom	Fs	c.2650del	p.(His884Thrfs*3)	32	Fs	c.2650del	p.(His884Thrfs*3)	32	Present
K	16,17	Comp het	Fs	c.2851del	p.(Glu951Lysfs*66)	23.8	Fs	c.3167dup	p.(Gly1057Argfs*11)	21.8	Present
L	18,19,20	Hom	Fs	c.3155dup	p.(Arg1053Thrfs*15)	22.7	Fs	c.3155dup	p.(Arg1053Thrfs*15)	22.7	Present
M	21	Hom	Non	c.3175C > T	p.(Arg1059*)	37	Non	c.3175C > T	p.(Arg1059*)	37	Khan^b^
N	22	Hom	Non	c.3175C > T	p.(Arg1059*)	37	Non	c.3175C > T	p.(Arg1059*)	37	Present
O	23	Hom	Fs	c.3346del	p.(Glu1116Asnfs*4)	33	Fs	c.3346del	p.(Glu1116Asnfs*4)	33	Present
P	24,25,26	Hom	Non	c.3514C > T	p.(Gln1172*)	38	Non	c.3514C > T	p.(Gln1172*)	38	Present
Q	27	Comp het	Mis	c.3698G > T	p.(Cys1233Phe)	29.1 (0.809)	Mis	c.4498C > T	p.(Arg1500Trp)	26 (0.606)	Khan^b^
R	28,29,30	Hom	Non^d^	c.4183_4185delinsAT	p.(Leu1395*)	34	Non^d^	c.4183_4185delinsAT	p.(Leu1395*)	34	Khan^b^
S	31	Hom	Non	c.4261C > T	p.(Gln1421*)	39	Non	c.4261C > T	p.(Gln1421*)	39	Present
T	32,33	Hom	Fs	c.4436del	p.(Pro1479Leufs*45)	35	Fs	c.4436del	p.(Pro1479Leufs*45)	35	Present
U	34,35	Hom	Non	c.4440C > G	p.(Tyr1480*)	40	Non	c.4440C > G	p.(Tyr1480*)	40	Present

*Note*: *ZNF142* variants are described using the NM_001105537.2 (GRCh37/hg19) transcript.

Abbreviations: Comp het, compound heterozygous; Fs, frameshift; Fam, family identification; Hom, homozygous; Non, nonsense; Mis, missense.

^a^ The individuals (Ind) from the same family are grouped together.

^b^ Reference 20.

^c^ Reference 21.

^d^ The variant was described as c.[4183del;4185G > A] *in cis* in the original publication (20). #, REVEL scores are also calculated for the missense variants.

Most of the variants (23/27) were predicted to result in a truncated protein: eight single‐nucleotide deletions, two two‐nucleotide deletions, two single‐nucleotide duplications, one indel, and 10 nonsense variants (Table 2). There were only four missense variants: two were identified in a compound heterozygous individual (individual 27) and two were *in trans* with protein‐truncating variants (Table 2). Three of the missense variants were predicted to change amino acid residues located within the ZF motifs (Figure 2), and none were predicted to affect splicing. Three protein‐truncating variants were recurrent: c.1252C > T, p.(Arg418*) in two families (family E, individuals 7 and 8; family F, individuals 9 and 10); c.1906C > T, p.(Arg636*) in two families (family H, individuals 12 and 13; family I, individual 14); and c.3175C > T, p.(Arg1059*) in two individuals (21 and 22). Only one frameshift variant (c.4436del, p.[Pro1479Leufs*45], individuals 32 and 33) was predicted to escape NMD in line with the 55‐bp rule, as it introduces a PTC within the final 55 bp of the penultimate exon 9 (Table S3). Most variants were either absent or present in an extremely low frequency (<0.0005%) in control populations (gnomAD; https://gnomad.broadinstitute.org/). Only six variants were present with a frequency of 0.0005%–0.06% (Table S3). All the variants had a CADD score (PHRED) higher than 20, except for the missense variant c.2288C > G in individual 1 (Table 2, Table S3). The REVEL scores of the compound heterozygous missense variants (c.3698G > T and c.4498C > T) in individual 27 were above 0.5, while the two missense variants (c.2288C > G and c.3885C > A in individuals 1 and 14, respectively) *in trans* with protein‐truncating variants had REVEL scores below 0.5 (Table 2, Table S3).

### 3.3 Protein modeling of the missense variants

**FIGURE 3:**
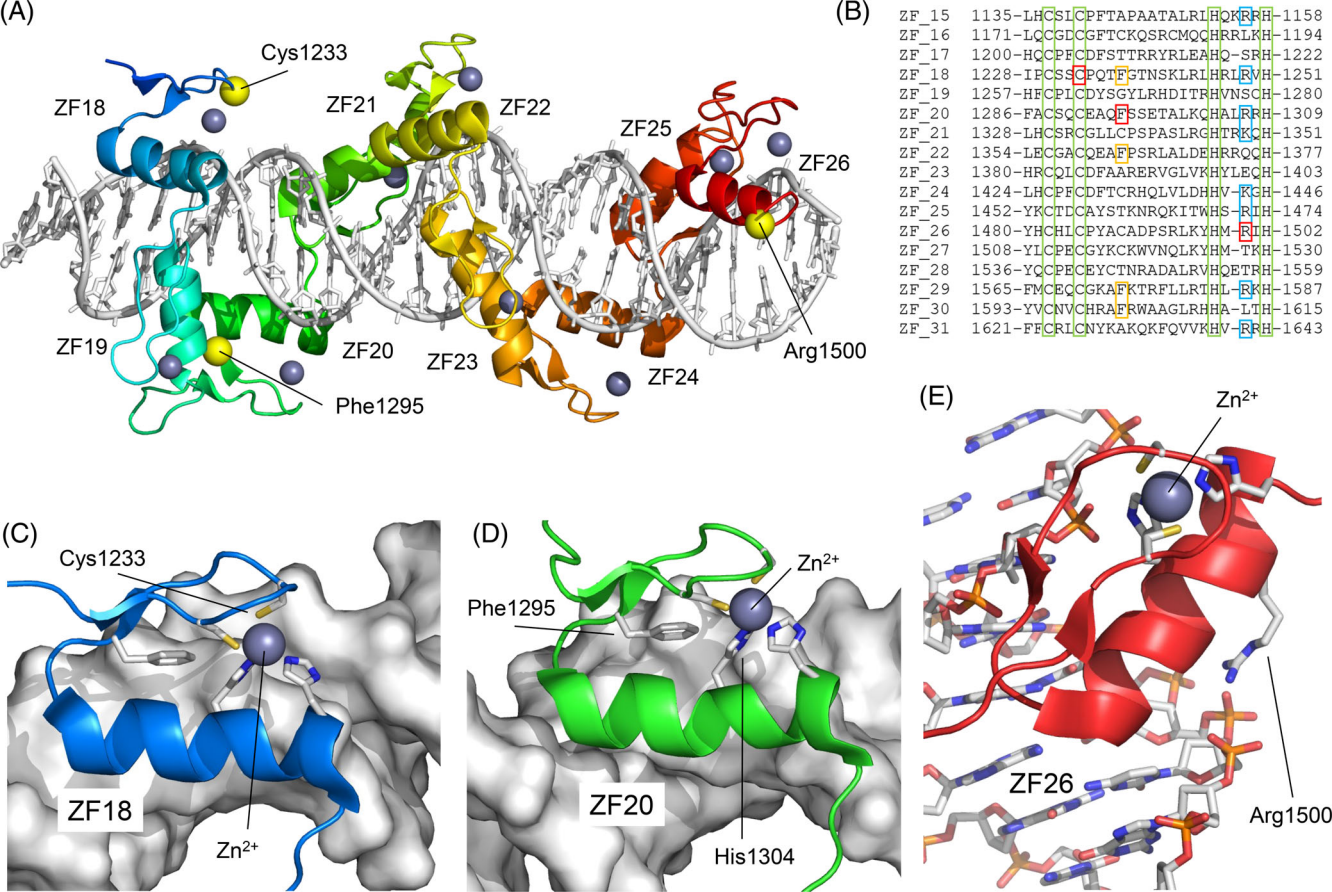
Structural model of the missense variants of *ZNF142*. (A) Structural model of ZF motifs 18–26 (ZF18 ‐ ZF26) of the protein and their interaction with DNA. Zn^2+^ ions are represented as gray spheres. The positions of the residues Cys1233, Phe1295, and Arg1500 are indicated. (B) Multiple sequence alignment of the 17 C‐terminal ZF motifs. The positions of Cys1233 (ZF18), Phe1295 (ZF20), and Arg1500 (ZF26) are shown in red. In green, the Cys and His residues that coordinate the Zn^2+^ ion (among them, Cys1233). In yellow, positions homologous to Phe1295 that are conserved. In blue, conserved positions with positive electrostatic charge homologous to Arg1500. (C) Position of Cys1233 coordinating the Zn^2+^ ion. The Cys1233Phe substitution would completely disorder the structure of ZF18. (D) Position of Phe1295 stabilizing the position of His1304, which, in turn, coordinates the Zn^2+^ ion. (E) Position of Arg1500 associating with the DNA phosphate chain. The Arg1500Trp substitution would prevent the correct interaction of ZF26 with DNA [Colour figure can be viewed at wileyonlinelibrary.com]

To study the possible effect of the four missense variants on the structure or the function of the protein, we made a 3D structural model of the domains in which these variants are located. As the p.(Ser763Cys) variant is within an unstructured segment (Figure 2) it was not possible to generate a 3D model for this position. p.(Cys1233Phe), p.(Phe1295Leu), and p.(Arg1500Trp) variants are within the C‐terminal ZF motifs (Figure 2; Figure 3). The Cys1233 residue (ZF motif 18) is conserved in all ZF motifs and is directly involved in the coordination of the Zn^2+^ ion. Substitution of Cys1233 with Phe is predicted to prevent correct coordination of the Zn^2+^ ion, directly affecting the structural stability of the motif. Similarly, Phe1295 (ZF motif 20) is involved in the positioning of His1304, which functions in the coordination of the Zn^2+^ ion. Substitution of Phe1295 with Leu is also expected to destabilize the conformation of the motif due to local structural rearrangement. Arg1500 (ZF motif 26) with a positive electrostatic charge is in contact with the negatively charged phosphate chain of the DNA molecule. Its replacement by a hydrophobic amino acid such as Trp is not likely to affect the local structure of the motif, but it is predicted to affect the interaction with DNA.

## 4 DISCUSSION

In this study, we describe the phenotype and genotype of 26 previously unpublished individuals from 16 unrelated families in comparison with nine previously published individuals from five families. The major features of the current cohort include varying degrees of ID (23/26, 89%), language impairment with variable severity (26/26, 100%), delay in developmental milestones (22/26, 85%), seizures (17/26, 65%), hypotonia (10/19, 52%), behavioral challenges (12/22, 55%) and a movement disorders (9/25, 36%). Even though the clinical features of the current cohort overlap with those previously reported,^20^, ^21^ there are discrepancies between the frequencies of specific features: Hypotonia was observed in fewer individuals in previous publications (3/9, 33%), while the frequencies of seizures (7/9, 78%) and movement disorders (5/9, 56%) were higher. The two siblings reported by Kameyama et al.^21^ did not have any movement disorders, while the frequency of dystonia (43%), tremor (57%), and ataxia (57%) was much higher in the families reported by Khan et al.^20^ compared to the frequencies observed in the current cohort (20%, 16%, and 28%, respectively). Similarly, the frequency of behavioral challenges, such as ASD, ADD/ADHD, anxiety, and aggressivity, was 55% in the current cohort, while previous publications did not underline such features. These differences are likely to be due to the small numbers of the previously reported individuals. This stresses the importance of investigating larger cohorts to understand the phenotypic spectrum of rare genetic disorders. Similarly, the sex bias towards females (six females, one male) observed in the previous publication^20^ was not verified in the current cohort where the female‐to‐male ratio is 3:2.

In the first publication linking *ZNF142* variants to a NDD, facial features of the seven individuals were not emphasized and clinical pictures were not included,^20^ while in the second publication, flat nasal bridge, micrognathia, and prominent eyes were recognized in the two siblings.^21^ Three dysmorphologists from our group independently inspected the facial pictures of these siblings and 12 individuals from the current cohort, and recognized hypertelorism and wide nasal bridge, telecanthus, thick eyebrows with lateral sparseness, facial hypotonia, and everted lower lip vermilion in more than half of the 14 individuals (Figure 1, Table S1). Furthermore, thick nasal alae, uplifted/large earlobe, and downturned corners of the mouth were also recurrent features (Table S1). Describing the facial morphology of individuals with a rare genetic disorder is often challenging as it is not always possible to obtain high‐quality clinical pictures and the features are not always evaluated independently by experienced dysmorphologists using standard terms. The above‐mentioned features should be reevaluated in the clinics by the referring clinicians, but the current findings suggest that individuals with biallelic *ZNF142* variants have some similar facial features.

Pathogenic variants in KRAB‐ZNF genes are likely to affect mainly the higher cortical functions, while the large poly‐ZNF subfamily of ZNFs is associated with increased risk of brain malformations.^3^, ^27^, ^28^, ^29^ This may be supported by brain imaging of the individuals with *ZNF142* variants, where no recognizable patterns or gross malformations were detected, even though delayed myelination and thinning of corpus callosum were recurrent. Little is known regarding the specific function of ZNF142, but other KRAB‐ZNF proteins are shown to be involved in chromatin remodeling, a key process reported to be altered in several NDDs,^30^ and modulation of transcription.^2^, ^3^, ^31^ ZNF142 is expressed in multiple tissues in humans, and RNA‐sequencing revealed an abundant expression of *ZNF142* in the developing brain.^32^ Of note, in concordance with the clinical features of the individuals with biallelic *ZNF14*2 variants, expression levels were high in the cerebellum and basal ganglia, brain structures involved in muscle coordination, and associated with movement disorders when affected postnatally, and in the temporal lobe, where the two language centers, Broca's and Wernicke's areas, are located.^32^ It has also been suggested that the lack of mouse orthologs of certain ZNFs associated with NDDs could indicate that they are responsible for higher cognitive function^13^, ^14^; however, this is not the case for ZNF142 which has a mouse ortholog.^33^

Most of the variants detected in this cohort were predicted as protein‐truncating variants and only four were missense variants, and interpretation of their effect can therefore be challenging. The p.(Ser763Cys) and p.(phe1295Leu) variants of individuals 1 and 14, respectively, were *in trans* with a truncating variant, and may be hypomorphic alleles. Individual 27 was the only person compound heterozygous for two missense variants (p.(Cys1233Phe) and p.(Arg1500Trp)). All three individuals (1, 14, and 27) had a somewhat milder phenotype compared to the individuals who are homozygous or compound heterozygous for truncating variants: They had normal or low average IQ or mild ID, and mildly affected language and motor development. Movement disorders were mild or absent, and likewise, the seizures were absent or treatable. The missense variant in individual 14 (*in trans* with a truncating variant) affects the local structure of a single ZF motif and may result in a partly functioning protein, which may explain the milder phenotype. In individual 1, the missense variant (also *in trans* with a truncating variant) could not be modeled but may also lead to a partially functioning protein in line with the milder phenotype. The two missense variants in individual 27 are predicted to affect a DNA‐ZF motif interaction and the local structure of a single ZF motif, respectively, and this may be an explanation of the milder phenotype. Notably, individuals 32 and 33 were homozygous for the p.(Pro1479Leufs*45) frameshift variant predicted to escape NMD and they also presented with a mild phenotype. However, the milder phenotype observed in individuals 12 and 13, who are compound heterozygous for p.(Arg636*) and p.(Leu1245Phefs*4) cannot be explained as these variants are neither predicted to escape NMD, nor did the nucleotide context of the variants suggest that the translation machinery would readthrough the PTCs.^34^ Nonetheless, due to the small sample size of the current cohort and the lack of experimental information regarding the function or localization of ZNF142, it is not possible to speculate on the exact effect of a defective ZNF142 on the phenotype.

In conclusion, the combined clinical data of 35 individuals suggest that biallelic variants in *ZNF142* result in a syndromic NDD most often including mild to moderate ID with variable degrees of delay in language and motor development, early‐onset seizures, behavioral challenges, hypotonia, and movement disorders with variable severity and recurrent facial features such as hypertelorism and wide nasal bridge, telecanthus, thick eyebrows with lateral sparseness, facial hypotonia, and everted lower lip vermilion. Further studies and larger cohorts are warranted to understand the pathophysiology and clinical features of this disorder.

## AUTHOR CONTRIBUTIONS

Conceptualization: Rikke S. Møller, Zeynep Tümer.

Data curation—genetic and clinical investigations: Nazanin A. Mohammadi, Marcello Niceta, Rauan Kaiyrzhanov, Chadi Al Alam, Viola Alesi, Valérie Benoit, Kailash P. Bhatia, Tatjana Bierhals, Christian M. Boßelmann, Julien Buratti, Bert Callewaert, Berten Ceulemans, Perrine Charles, Matthias De Wachter, Mohammadreza Dehghani, Maria Lisa Dentici, Erika D'haenens, Martine Doco‐Fenzy, Michaela Geßner, Cyrielle Gobert, Ulviyya Guliyeva, Tobias B. Haack, Trine B. Hammer, Tilman Heinrich, Maja Hempel, Theresia Herget, Ute Hoffmann, Judit Horvath, Henry Houlden, Boris Keren, Christina Kresge, Candy Kumps, Damien Lederer, Alban Lermine, Francesca Magrinelli, Reza Maroofian, Mohammad Y.V. Mehrjardi, Mahdiyeh Moudi, Amelie J. Müller, Anna J. Oostra, Beth A. Pletcher, Francesca Clementina Radio, Shanika Samarasekera, Marco Tartaglia, Kristof Van Schil, Julie Vogt, Evangeline Wassmer, Juliane Winkelmann, Maha S. Zaki, Michael Zech, Holger Lerche, Rikke S. Møller, Zeynep Tümer.

Data analysis: Maria B. Christensen, Amanda M. Levy, Zeynep Tümer.

Project administration: Maria B. Christensen, Amanda M. Levy, Zeynep Tümer.

Visualization: Paulino Gomez‐Puertas, David Ros‐Pardo, Amanda M. Levy.

Preparation of the original draft: Maria B. Christensen, Amanda M. Levy, Zeynep Tümer.

Reviewing and commenting the manuscript: All the authors.

Fine tuning and final editing of the manuscript: Zeynep Tümer, Amanda M. Levy.

## CONFLICT OF INTEREST

The authors declare no conflict of interest.

## Supporting information


**TABLE S1** Independent evaluation of facial features by three dysmorphologists from our group

**TABLE S2** Detailed description of the phenotypes with genotypes

**TABLE S3** Variant pathogenicity

## Data Availability

The data that support the findings of this study are available on request from the corresponding author. The data are not publicly available due to privacy or ethical restrictions.
