# Supplementary material for: Biallelic variants in ZNF142 lead to a syndromic neurodevelopmental disorder
Source: Clin Genet. 2022 Jun 8;102(2):98–109. doi: 10.1111/cge.14165 (PMC9546172; doi:10.1111/cge.14165)
Supplement: Supplementary file 1 — TABLE S1 Independent evaluation of facial features by three dysmorphologists from our group [file CGE-102-98-s001.docx]

|  | **Evaluation 1 (MLD)** | | **Evaluation 2 (FCR)** | | **Evaluation 3 (DL)** | |
| --- | --- | --- | --- | --- | --- | --- |
| **Dysmorphic features (HPO)** | **Individuals** | **Total^a^** | **Individuals** | **Total^a^** | **Individuals** | **Total^a^** |
| **Hypertelorism/wide nasal bridge (HP:0000316/HP:0000431)** | 2, 5, 6, 11, 15, 16, 17, 22, 23, 31, 34, 35, K1, K2 | 14/14 (100%) | 2, 5 (in childhood), 6 (in childhood), 11, 16, 17, 22, 23, 34, 35, K1, K2 | 12/14 (86%) | 5, 6, 11, 16, 17, 22, 23, 31, 34, 35, K1, K2 | 12/14 (86%) |
| **Telecanthus (HP:0000506)** | 5, 22, 23, 34, 35, K1, K2 | 7/14 (50%) | 5, 17, 22, 23, 34, 35, K1, K2 | 8/14 (57%) | 5, 6 (in childhood),17, 22, 23, 34, 35, K1, K2 | 9/14 (64%) |
| **Thick eyebrows (HP:0000574) with lateral sparseness (HP:0005338)** | 2, 5, 11, 16, 34, 35, K2 | 7/14 (50%) | 2, 5, 6, 11, 16, 17, 34, 35, K2 | 9/14 (64%) | 2, 5, 11, 16, 17, 34, 35, K2 | 8/14 (57%) |
| **Everted lower lip vermilion (HP: 0000232)** | 2, 5, 17, 22, 23, 34, 35, K1, K2 | 9/14 (64%) | 2, 5, 11, 17, 22, 31, 34, 35, K1, K2 | 10/14 (71%) | 2, 5, 11, 17, 22, 31, 34, 35, K1, K2 | 10/14 (71%) |
| **Facial hypotonia (HP:0000297)** | 5, 17, 22, 31, 35, K1, K2 | 7/14 (50%) | 5,6, 17, 22, 31, 34, 35, K2 | 8/14 (57%) | 2, 5, 17, 22, 31, 34, 35, K1, K2 | 9/14 (64%) |
| **Downturned corners of mouth (HP:0002714)** | 2, 15, 17, 22, 31, 35, K1, K2 | 8/11 (73%) | 2, 17, 22, 35, K1, K2 | 6/11 (54%) | 2, 15, 31, 35 | 4/11 (36%) |
| **Thick nasal alae (HP:0009928)** | 16, 17, 22, 34, 35, K1, K2 | 7/10 (70%) | 5, 16, 17, 22, 34, 35, K1, K2 | 8/10 (80%) | 16, 17, 22, 35, K1, K2 | 6/10 (60%) |
| **Uplifted/large earlobe (HP:0009909/HP:0009748)** | 6, 17, 22, 35, K1 | 5/10 (50%) | 6, 22, 23, 31, 35 | 5/10 (50%) | 6, 16, 22, 23, 35 | 5/10 (50%) |

**Supplementary Table S1 – Independent evaluation of facial features by three dysmorphologists from our group**

a, The denominator indicates the number of individuals, where a given feature could be evaluated. K1 and K2, individuals reported by Kamemaya et al. (21)
